# Biomechanical Performance of Total Wrist Arthrodesis Plates With and Without Arthrodesis of the Carpometacarpal Joint

**DOI:** 10.1177/15589447231198263

**Published:** 2023-10-07

**Authors:** David H. Owen, Dongyang Wang, Xu Cong, Cameron Mowbray, Diana M. Perriman, Chris J. Roberts, Paul N. Smith, Herwig Drobetz, David Ackland

**Affiliations:** 1 Canberra Hospital, ACT, Australia; 2 Australian National University Medical School, Canberra, ACT, Australia; 3 The University of Melbourne, VIC, Australia; 4 Lismore Base Hospital, NSW, Australia; 5 Bond University, Robina, QLD, Australia

**Keywords:** carpometacarpal joint, carpal, metacarpal, total wrist arthrodesis, total wrist fusion, biomechanical

## Abstract

**Background:**

It is unknown whether total wrist arthrodesis (TWA) should be performed with or without arthrodesis of the carpometacarpal joint (CMCJ). The aim of this study is to compare CMCJ-spanning TWA plates using 3D printed wrist arthrodesis model with and without arthrodesis of the CMCJ.

**Methods:**

Total wrist arthrodesis plates mounted to 3D printed models were tested under a 4-N bending load at 4 Hz for 50 000 cycles, increased by 15% every 10 000 cycles until failure.

**Results:**

Plates with arthrodesis CMCJ were stiffer and failed at a significantly greater load and number of cycles than plates mounted to models without CMCJ arthrodesis. The Synthes stainless steel locking TWA plate performed better than the Trimed plate applied to the model without CMCJ arthrodesis and the Acumed plate applied to the model with CMCJ arthrodesis. Based on these findings, we recommend arthrodesis of the CMCJ in TWA.

**Conclusions:**

Incorporation of the CMCJ in TWA may protect against plate failure. If arthrodesis of the CMCJ is not performed, plate removal should be considered before breakage occurs.

**Level of Evidence:**

IV

## Introduction

Total wrist arthrodesis (TWA) is the surgical treatment of choice for patients with advanced disease of the wrist that is not amenable to motion-preserving procedures.^1^ Dorsally applied precontoured plates have gained widespread popularity for TWA and are supported by a large body of evidence demonstrating excellent clinical performance.^2-6^ Precontoured locking TWA plates have several advantages over alternatives.^7,8^ These include a low-profile, tapered shape that minimizes extensor tendon irritation and anatomical contour that facilitates application directly to the radius and carpal bones.

Arthrodesis of the carpometacarpal joint (CMCJ) in TWA is controversial.^9-12^ There are three approaches to the CMCJ in wrist arthrodesis: (1) preparation of the joint for arthrodesis and bridging with a plate, in which case the plate is typically not removed^10^; (2) preservation of the CMCJ and spanning of the joint, in which case there is no attempt at arthrodesis, and many authors have described leaving the plate in, while others advocate removing it^11^; and finally (3) preservation of the CMCJ and application of a non-spanning plate, without arthrodesis of the CMCJ.^9,12^

Superiority of either approach to the CMCJ in TWA has not been determined. Arthrodesis of the CMCJ has been hypothesized to reduce force on the plate, lowering the chances of plate failure and reducing the need for plate removal, which many surgeons consider problematic.^2^ Some authors advocate for arthrodesis of the CMCJ in certain populations, such as in heavy manual workers.^13^ It is also speculated that the CMCJ may become arthritic and painful due to excessive loading after TWA, which is prevented by successful arthrodesis.

Bridging the CMCJ without arthrodesis preserves some motion after the plate is removed. It is estimated that the third CMCJ has 7° of flexion/extension.^14^ It is unknown whether this is clinically important. If plates are not removed, prolonged loading due to movement at the CMCJ may result in plate breakage or loosening.

Bridging the CMCJ without arthrodesis avoids arthrodesis of the CMCJ, which is prone to nonunion and represents a significant proportion of postoperative complications.^11^ Neither approach to the treatment of the CMCJ in TWA has been shown to be superior.^10-12,15^

Construct studies examining TWA have compared Steinmann pins to 3.5 locking compression plates, 3.5-mm dynamic compression plates to 2.7-/3.5-mm Synthes (Warsaw, Indiana) titanium TWA plates (Synthes Ti), and 2.7/3.5 Synthes stainless steel (Synthes SS) plates to non–CMCJ-spanning TWA plates.^16-18^ Morelli et al^16^ showed nonlocking dynamic compression plates and TWA plates were equivalent and performed better than Steinmann pins. Richards et al^18^ showed 3.5-mm dynamic compression plates performed similar to 2.7-/3.5-mm TWA plates in cadaveric wrists. Passin et al^17^ compared the performance of the Medartis (Basel, Switzerland) APTUS non–CMCJ-spanning plates to the 2.7/3.5 Synthes SS locking TWA plates and reported that the APTUS plate was superior; however, the loading conditions of each plate were not equivalent. No biomechanical study has examined treatment of the CMCJ in TWA.

We used low-force, high-frequency loading of a wrist arthrodesis model, with radiocarpal and intercarpal arthrodesis, with or without arthrodesis of the CMCJ, to test plate performance. We hypothesized that TWA plates mounted to models with arthrodesis of CMCJ would outperform those mounted to models without CMCJ arthrodesis.

## Materials and Methods

Six small bend-sized TWA plates from Acumed (Hillsboro, Oregon), Medartis, Stryker (Kalamazoo, Michigan), Synthes locking stainless steel (SS) and Synthes non-locking Titanium (Ti), and Trimed (Valencia, California) plates were evaluated. All plates were mounted in triplicate on 3D printed wrist models as described by Passin et al,^17^ adapted to examine arthrodesis of the CMCJ. A detailed description of screw configuration for each plate tested is given in Supplemental Material 1. Models were created from Standard Triangle Language files provided by Passin et al^17^ and adapted using Autodesk Tinkercad Online (tinkercad.com, Autodesk, San Rafael, California) and Autodesk Meshmixer v3.5.474 (Autodesk, San Rafael, California) and the build file produced using Stratasys Grabcad Print v1.47. Models were created using fused deposition modeling using acrylonitrile styrene acrylate thermoplastic filaments on a Stratasys F123 (Eden Prairie, Minnesota) 3D printer with a T14 tip, slice height 0.25 mm, 100% in-fill, 62 cm^3^ density, and 2.65-hour print time. This material has a modulus of elasticity and ultimate strength between human cancellous and cortical bone.^19-22^ In the CMCJ arthrodesis model, the radiocarpal joint, midcarpal joint, and CMCJ were one solid continuous structure (See Supplemental Material 2—wrist model with CMCJ arthrodesis). A 2-mm split and cut-out at the level of the CMCJ that allowed motion was used to represent a mobile CMCJ in the model without CMCJ arthrodesis (Supplemental Material 3—wrist model with mobile CMCJ). Motion of the CMCJ was therefore limited by bending of the plate alone.

**Figure 1.**
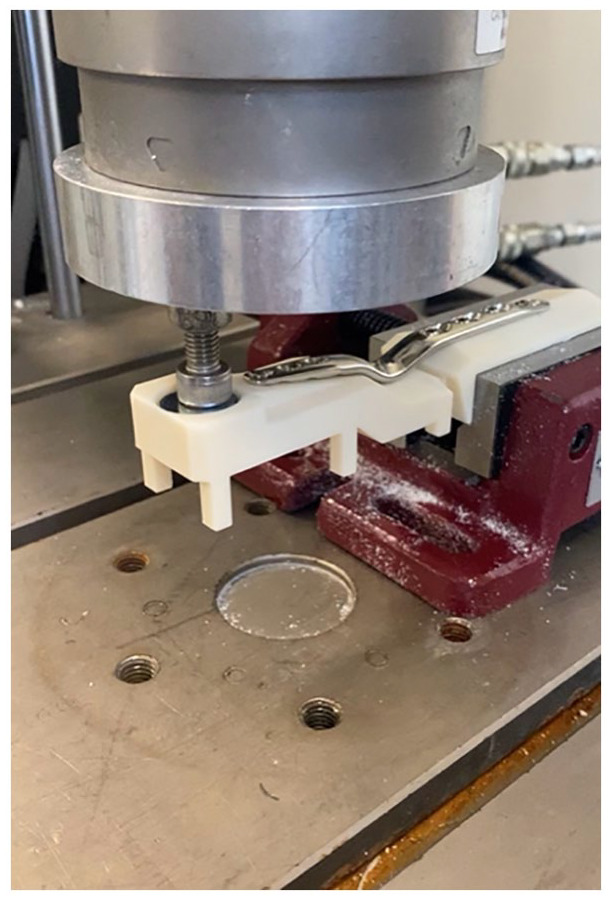
The experimental testing setup showing the Synthes locking stainless steel plate and fixation screws, load cell, load applicator, and 3D printed wrist model with arthrodesis of the carpometacarpal joint mounted in support clamp on the Instron machine base plate.

Plates were fixed to the models according to manufacturer instructions by an orthopedic surgeon (D.H.O.). The Acumed plate is designed to span between the radius and the second metacarpal; other plates are typically applied between the radius and the third metacarpal. A burr was used to modify the model to allow the Acumed plate to sit flush on the wrist model (Supplemental Material 4). A jig was used to ensure plates were mounted in an identical position. A bench-top vice fixed to a material-testing machine was used to secure the proximal end of the model (see Figure 1, Supplemental Material 5). An axial force was applied perpendicular to the axis of the metacarpal in a dorsal-palmer direction at a fixed position on the metacarpal head to create a bending force across the wrist model (Instron Model 3521; Parker Hydraulics, Warwick, UK, instrumented with a 10-kN uniaxial load cell, Instron 67918; Instron, Buckinghamshire, UK) at 4 Hz × 0.1 N to 70 N for 50 000 cycles. Loading was then increased by 15% every 10 000 cycles until failure.^23^ Displacement and load were sampled at 50 Hz. Construct stiffness (S) was calculated using the formula S = F/δ, where F is the total load and δ the bending deflection. Failure was defined as displacement greater than 15 mm or fracture of any part of the construct.^17^ The number of cycles to failure, load at failure, and the mechanism of failure were recorded.

Mann-Whitney U test was used to assess the difference between plates mounted to models with and without CMCJ arthrodesis. Kruskal-Wallis single-way analysis of variance for nonparametric data was used to analyze differences between groups. Dunn’s pairwise comparison with Bonferroni adjustment was used to assess statistical difference (*P* < .05) between plate types. Statistical analysis was performed using Stata 17 software (StataCorp. 2021. Stata Statistical Software: Release 17; StataCorp LLC, College Station, Texas).

## Results

### Cycles to Failure

**Figure 2.**
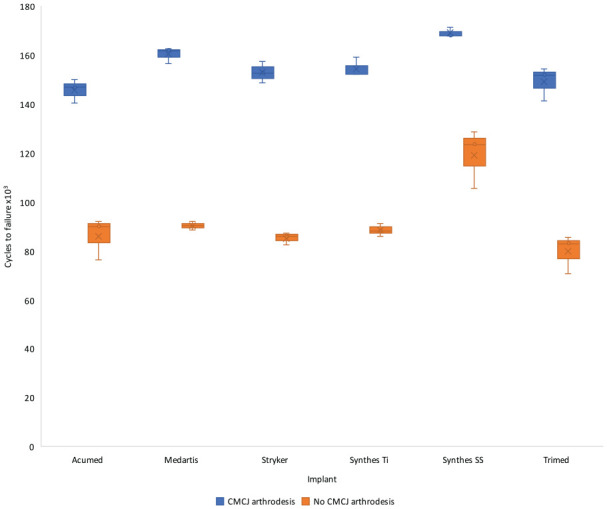
Cycles to construct failure grouped by plate with or without arthrodesis of the CMCJ, shown graphically. Plates mounted to models were loaded at 4 Hz × 0.1 N to 70 N for 50 000 cycles. Loading was then increased by 15% every 10 000 cycles until failure. Data are presented with whiskers representing minimum and maximum values, bar representing interquartile range, line showing median, and cross showing mean. *Note.* CMCJ = carpometacarpal joint; Ti = titanium; SS = stainless steel.

Plates mounted to models without CMCJ arthrodesis failed at 60% of the cycles required for failure of models with CMCJ arthrodesis, median of 91 589 cycles (interquartile range [IQR] 85 473-92 055) versus 152 745 (IQR 149 999-161 901), *P* < .001. In models without CMCJ arthrodesis, the Synthes SS plate performed significantly better than the Trimed plate (*P* = .01) (Table 1). In the model with CMCJ arthrodesis, the Synthes SS plate performed significantly better than the Acumed plate (*P* = .01) (Figure 2).

**Table 1.** Cycles to Construct Failure Grouped by Plate With or Without Arthrodesis of the CMCJ.

Implant	No CMCJ arthrodesis Cycles (25%-75% IQR)	CMCJ arthrodesis Cycles (25%-75% IQR)
Acumed	90 000 (76 472-92 055)	146 963 (140 396-140 999)
Medartis	90 528 (88 726-92 301)	161 901 (156 678-162 630)
Stryker	85 977 (82 647-87 376)	152 745 (148 716-157 678
Synthes SS	123 503 (105 804-128 608)	168 074 (168 061-171 254)
Synthes Ti	88 322 (86 009-91 091)	152 331 (152 318-159 286)
Trimed	83 122 (70 593-85 473)	152 041 (141 492-154 609)

*Note.* The median number of cycles to failure is given with interquartile range (IQR). The number of samples in each group is 3. CMCJ = carpometacarpal joint.

### Load at Failure

**Figure 3.**
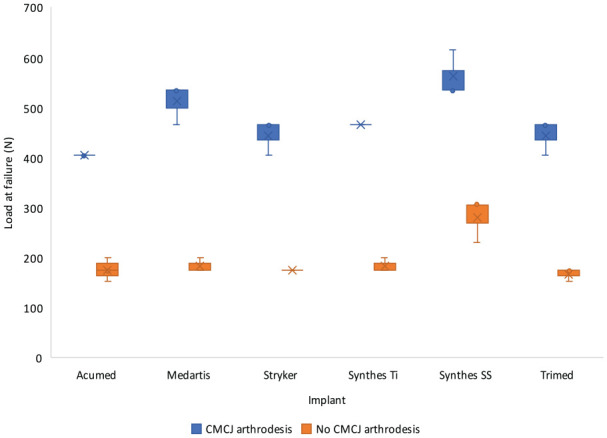
Load to construct failure shown graphically grouped by plate with or without arthrodesis of the CMCJ. Plates mounted to models were loaded at 4 Hz × 0.1 N to 70 N for 50 000 cycles. Loading was then increased by 15% every 10 000 cycles until failure. Each group includes three samples. Data are presented with whiskers representing minimum and maximum values, bar representing interquartile range, line showing median, and cross showing mean. *Note.* CMCJ = carpometacarpal joint; Ti = titanium; SS = stainless steel.

Failure of TWA plates mounted to models without CMCJ arthrodesis occurred at a median force of 87.45 N (IQR 87.5-100.5), which was significantly lower than the 465.2 N (IQR 404.5-535.0, *P* = .012) used on the plates mounted to models with CMCJ arthrodesis (Table 2 and Figure 3). The Synthes SS plate failed at a higher load than the Trimed plate (*P* = .026), mounted to the model without CMCJ arthrodesis. The Synthes SS plate also failed at a higher load than the Acumed plate, mounted to the model with CMCJ arthrodesis (*P* = .012). There was no statistical difference between other plates.

**Table 2.** Load at Construct Failure Grouped by Plate With or Without Arthrodesis of the CMCJ.

Implant	No CMCJ arthrodesis Load N (25%-75% IQR)	CMC arthrodesis Load N (25%-75% IQR)
Acumed	87.5 (6.0-100.5)	404.6 (404.6-404.6)
Medartis	100.5 (87-100.5)	535.0 (465.2-535.0)
Synthes SS	306.0 (231.3-306.0)	535.0 (535.0-615.3)
Synthes Ti	87.5 (87.5-100.5)	465.2 (465.2-465.2)
Synthes	87.5 (87.5-87.5)	465.2 (404.6-465.2)
Trimed	87.5 (76.0-87.5)	465.2 (404.6-465.2)

*Note.* The median load at failure is given in Newtons (N) with interquartile range (IQR). The number of samples in each group is 3. CMCJ = carpometacarpal joint.

### Plate Stiffness

**Figure 4.**
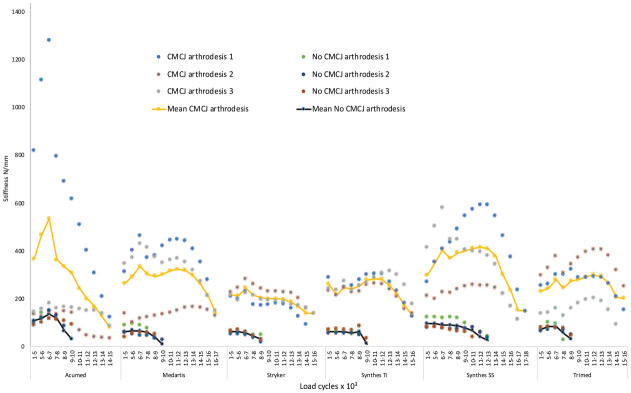
Plate stiffness is shown graphically with load cycle grouped by plate type. The mean plate stiffness (N/mm) is plotted every 1000 load cycles and shown with dot for each plate. The mean construct stiffness is plotted with a solid yellow line for plates mounted to a model with arthrodesis of the CMCJ and a solid black line for plates mounted to models without arthrodesis CMCJ. *Note.* CMCJ = carpometacarpal joint; Ti = titanium; SS = stainless steel.

The median stiffness of constructs at 6000 to 7000 cycles was 76.2 N/mm (IQR 57.7-89.4) without CMCJ arthrodesis compared with 332.6 N/mm (IQR 160-406.4) with CMCJ arthrodesis (*P* = .0001). There was no difference in stiffness between the plate types mounted to models without CMCJ arthrodesis (*P* > .05) and those mounted to models with CMCJ arthrodesis at 6000 to 7000 cycles (*P* > .05) (Figure 4).

### Mode of Failure

**Figure 5.**
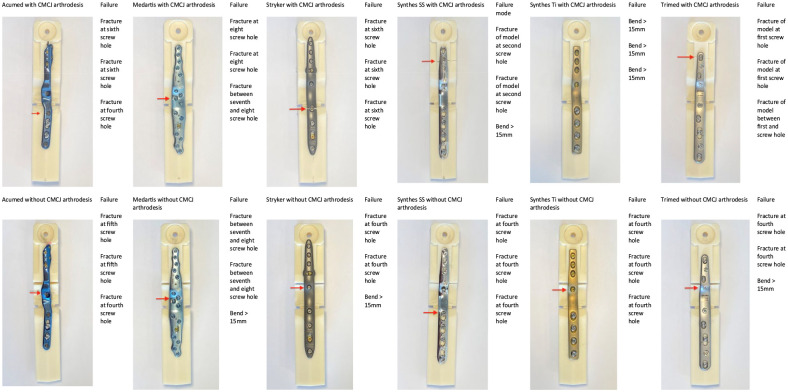
Mode of construct failure. The photographs provided show the most common mode of plate failure for each plate failure (n = 3). A red arrow indicates the location of the plate or model failure. The Synthes Ti plate failed by bending deformation great than 15 mm (not indicated by a red arrow). *Note.* CMCJ = carpometacarpal joint; Ti = titanium; SS = stainless steel.

Plates mounted to models without CMCJ arthrodesis generally failed at the fourth- or fifth-most distal screw hole as shown in Figure 5. The Medartis TWA plate generally failed around the seventh screw hole when mounted to both wrist models. Failure of the wrist model without implant failure was observed in Synthes SS and Trimed plates mounted to models with CMCJ arthrodesis.

## Discussion

This biomechanical study aimed to examine plate failure with and without arthrodesis of the CMCJ in TWA. It showed that arthrodesis of the CMCJ results in a stronger construct that resists more load cycles before failure regardless of the plate used. Construct stiffness was also significantly increased by arthrodesis of the CMCJ. These findings indicate that CMCJ arthrodesis protects the plate. There were few differences between plate types in cycles to failure, load at failure, and construct stiffness. The Synthes SS plate outperformed the Trimed plate when mounted to the model without CMCJ arthrodesis. The most common site of failure was at the fourth hole in the Trimed plate, which was located close to the CMCJ in the model.

The Synthes SS plate outperformed the Acumed plate, which failed at a lower number of cycles and lower load when mounted to the wrist model with CMCJ arthrodesis. The Acumed plate had a high variation in stiffness when compared to other plates. This may have been due to the shape of the plate, which is designed to conform to the radial aspect of the second metacarpal as opposed to the dorsal aspect of either the second or third metacarpal. Failure of the Acumed plate was most common at the fifth screw hole.

The wrist model with CMCJ arthrodesis failed before the Trimed (3/3 samples) and Synthes SS (2/3 samples) plates failed. In these cases, the model was the limiting factor in the assessment of these plates. This may reflect an interaction between the model and the plate where, for example, a better fit of the plate to the model or differing plate length may promote model failure before plate breakage.

Hardware breakage following fracture fixation and joint arthrodesis generally indicates nonunion.^24^ This study indicates that TWA without arthrodesis of the CMCJ risks plate breakage at a lower force and number of load cycles. The clinical implication of this finding is if the CMCJ is bridged, plates should be removed to prevent breakage with long-term loading. Conversely, if union of the CMCJ is obtained, plates are relatively protected from extended repetitive loading.

This investigation was a biomechanical study and therefore limited in its conclusions applicable to clinical practice. Other limitations that should be considered are that 6 plates from each manufacturer were tested under 2 different conditions. These numbers are comparable to previous studies but insufficient to establish firm statistical conclusions.^16-18,25^ The use of 3D printed wrist models had several advantages, including ease of manufacture, low cost, and standard mechanical properties that are comparable to bones.^17,19,20^ We choose models over cadaveric wrists for these reasons. It would have also been difficult to replicate arthrodesis of the radiocarpal in intercarpal joints using cadaveric wrists and control for differences in bone density between cadaveric specimens.^26^ Testing with additional axial and torsional forces experienced by the normal wrist would have made testing difficult and have added significant complexity to the loading conditions and analysis.

This biomechanical study demonstrates that arthrodesis of the CMCJ in TWA improves load-bearing characteristics of the plate. Successful arthrodesis of the CMCJ in TWA should reduce plate breakage and need for additional surgery. Additional high-quality clinical evidence is required to support this conclusion.

## Supplemental Material

sj-docx-1-han-10.1177_15589447231198263 – Supplemental material for Biomechanical Performance of Total Wrist Arthrodesis Plates With and Without Arthrodesis of the Carpometacarpal Joint Supplemental material, sj-docx-1-han-10.1177_15589447231198263 for Biomechanical Performance of Total Wrist Arthrodesis Plates With and Without Arthrodesis of the Carpometacarpal Joint by David H. Owen, Dongyang Wang, Xu Cong, Cameron Mowbray, Diana M. Perriman, Chris J. Roberts, Paul N. Smith, Herwig Drobetz and David Ackland in HAND

sj-docx-4-han-10.1177_15589447231198263 – Supplemental material for Biomechanical Performance of Total Wrist Arthrodesis Plates With and Without Arthrodesis of the Carpometacarpal Joint Supplemental material, sj-docx-4-han-10.1177_15589447231198263 for Biomechanical Performance of Total Wrist Arthrodesis Plates With and Without Arthrodesis of the Carpometacarpal Joint by David H. Owen, Dongyang Wang, Xu Cong, Cameron Mowbray, Diana M. Perriman, Chris J. Roberts, Paul N. Smith, Herwig Drobetz and David Ackland in HAND

sj-docx-5-han-10.1177_15589447231198263 – Supplemental material for Biomechanical Performance of Total Wrist Arthrodesis Plates With and Without Arthrodesis of the Carpometacarpal Joint Supplemental material, sj-docx-5-han-10.1177_15589447231198263 for Biomechanical Performance of Total Wrist Arthrodesis Plates With and Without Arthrodesis of the Carpometacarpal Joint by David H. Owen, Dongyang Wang, Xu Cong, Cameron Mowbray, Diana M. Perriman, Chris J. Roberts, Paul N. Smith, Herwig Drobetz and David Ackland in HAND

sj-stl-2-han-10.1177_15589447231198263 – Supplemental material for Biomechanical Performance of Total Wrist Arthrodesis Plates With and Without Arthrodesis of the Carpometacarpal Joint Supplemental material, sj-stl-2-han-10.1177_15589447231198263 for Biomechanical Performance of Total Wrist Arthrodesis Plates With and Without Arthrodesis of the Carpometacarpal Joint by David H. Owen, Dongyang Wang, Xu Cong, Cameron Mowbray, Diana M. Perriman, Chris J. Roberts, Paul N. Smith, Herwig Drobetz and David Ackland in HAND

sj-stl-3-han-10.1177_15589447231198263 – Supplemental material for Biomechanical Performance of Total Wrist Arthrodesis Plates With and Without Arthrodesis of the Carpometacarpal Joint Supplemental material, sj-stl-3-han-10.1177_15589447231198263 for Biomechanical Performance of Total Wrist Arthrodesis Plates With and Without Arthrodesis of the Carpometacarpal Joint by David H. Owen, Dongyang Wang, Xu Cong, Cameron Mowbray, Diana M. Perriman, Chris J. Roberts, Paul N. Smith, Herwig Drobetz and David Ackland in HAND
