# Supplementary material for: Biomechanical Performance of Total Wrist Arthrodesis Plates With and Without Arthrodesis of the Carpometacarpal Joint
Source: Hand (N Y). 2023 Oct 7;20(2):230–6. doi: 10.1177/15589447231198263 (PMC11833838; doi:10.1177/15589447231198263)
Supplement: sj-docx-1-han-10.1177_15589447231198263 – Supplemental material for Biomechanical Performance of Total Wrist Arthrodesis Plates With and Without Arthrodesis of the Carpometacarpal Joint [file sj-docx-1-han-10.1177_15589447231198263.docx]

| Plate: Acumed  Plate material: Titanium alloy | Screw configuration:   1. 2.3 locking screw (distal) 2. 2.3 locking screw 3. 2.3 locking screw 4. 2.3 non-locking screw 5. 3.5 locking screw 6. 3.5 locking screw 7. 3.5 non-locking screw 8. 3.5 non-locking screw 9. 3.5 locking screw (proximal) |
| --- | --- |
| Plate: Medartis  Plate material: Titanium alloy | Screw configuration:   1. 2.5mm locking screw (distal) 2. 2.5mm locking screw 3. 2.5mm locking screw 4. 2.5mm locking screw 5. 2.5mm locking screw 6. 2.5mm locking screw 7. 2.5mm locking screw 8. 2.5mm locking screw 9. 2.5mm locking screw 10. 2.5mm locking screw 11. 2.5mm locking screw 12. 2.5mm non-locking screw 13. 2.5mm locking screw 14. 2.5mm locking screw 15. 2.5mm locking screw 16. 2.5mm locking screw (proximal) |

| Plate: Stryker  Plate material: Titanium alloy | Screw configuration:   1. 2.7 locking screw (distal) 2. 2.7 locking screw 3. 2.7 locking screw 4. 2.7 non-locking screw 5. 2.7 locking screw 6. 3.5 locking screw 7. 3.5 locking screw 8. 3.5 non-locking screw 9. 3.5 locking screw (proximal) |
| --- | --- |
| Plate: Synthes SS  Plate material: stainless steel | Screw configuration:   1. 2.7mm non-locking screw (distal) 2. 2.7mm locking screw 3. 2.7mm locking screw 4. 2.7mm locking screw 5. 3.5mm locking screw 6. 3.5mm locking screw 7. 3.5mm locking screw 8. 3.5mm non-locking screw (proximal) |

| Plate: Synthes  Plate material: Titanium alloy | Screw configuration:   1. 2.7mm non-locking screw (distal) 2. 2.7mm non-locking screw 3. 2.7mm non-locking screw 4. 2.7mm non-locking screw 5. 3.5mm non-locking screw 6. 3.5mm non-locking screw 7. 3.5mm non-locking screw 8. 3.5mm non-locking screw (proximal) |
| --- | --- |
| Plate: Trimed  Plate material: stainless steel | Screw configuration:   1. 2.7mm non-locking (distal) 2. 2.7mm locking 3. 2.7mm non-locking 4. 2.7mm locking 5. 3.2mm locking 6. 3.2mm non-locking 7. 3.2mm non-locking 8. 3.2mm locking 9. 3.2mm non-locking (proximal) |

Supplementary material 1: Table showing plate type with screw configuration from most distal (1) to most proximal. Wrist models with CMCJ arthrodesis and mobile CMCJ had the same screw configuration.
