# Supplementary material for: Biomechanical Performance of Total Wrist Arthrodesis Plates With and Without Arthrodesis of the Carpometacarpal Joint
Source: Hand (N Y). 2023 Oct 7;20(2):230–6. doi: 10.1177/15589447231198263 (PMC11833838; doi:10.1177/15589447231198263)
Supplement: sj-docx-4-han-10.1177_15589447231198263 – Supplemental material for Biomechanical Performance of Total Wrist Arthrodesis Plates With and Without Arthrodesis of the Carpometacarpal Joint [file sj-docx-4-han-10.1177_15589447231198263.docx]

Supplementary Material 4 showing modification to the 3D printed wrist model to improve the fit of the Acumed plate. The Acumed plate is designed to fit to the dorsum and radial boarder of the second metacarpal. It also has an increased contour at the radial carpal joint level.


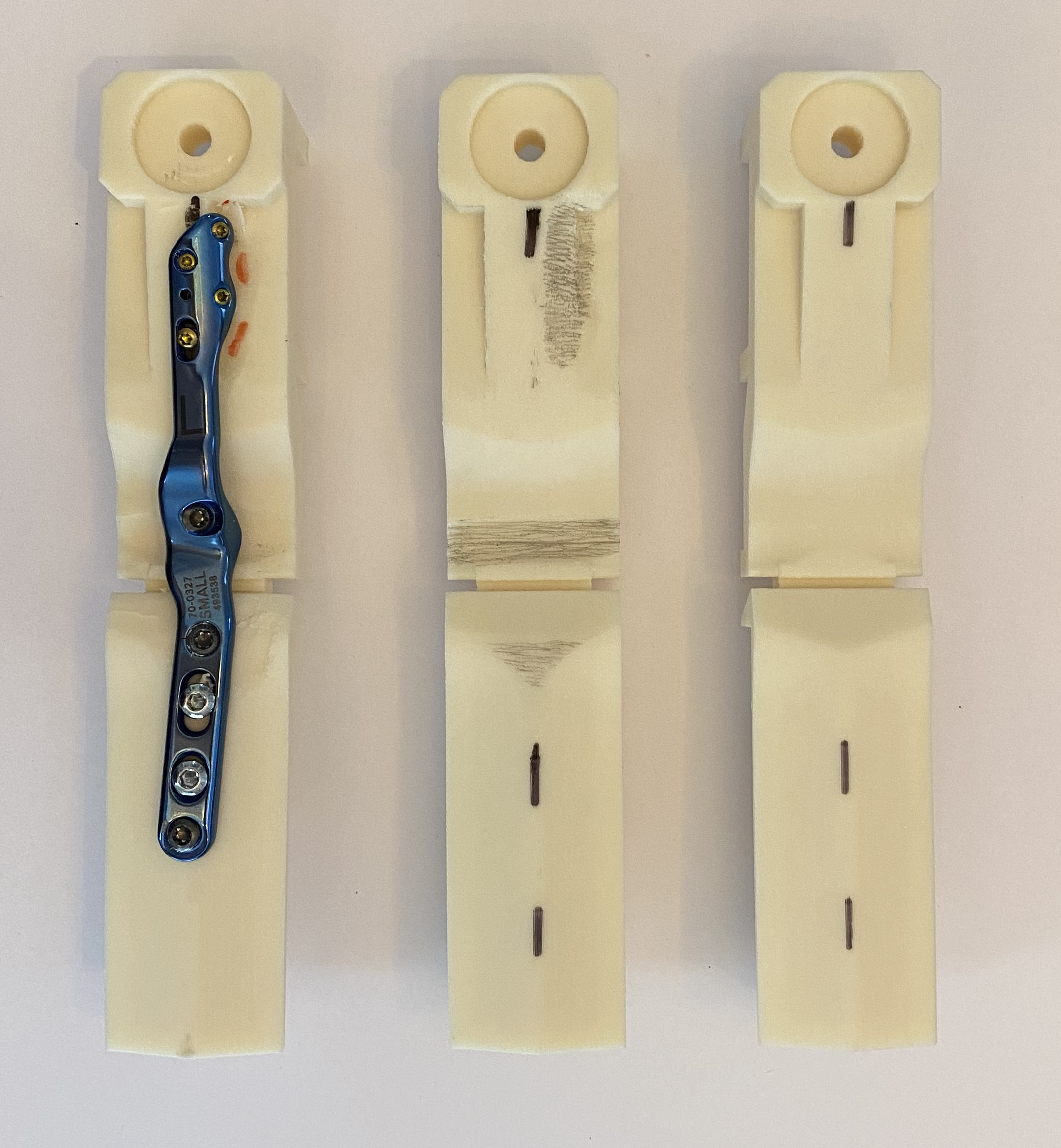


Anterior view of wrist and plate model

Left: Acumed plate applied to prepared model (with CMCJ arthrodesis, shown after testing)

Middle: Model showing areas shaded in grey lead pencil that are filed away to allow the plate to sit flush on the model.

Right: Wrist model without modifications for the Acumed plate


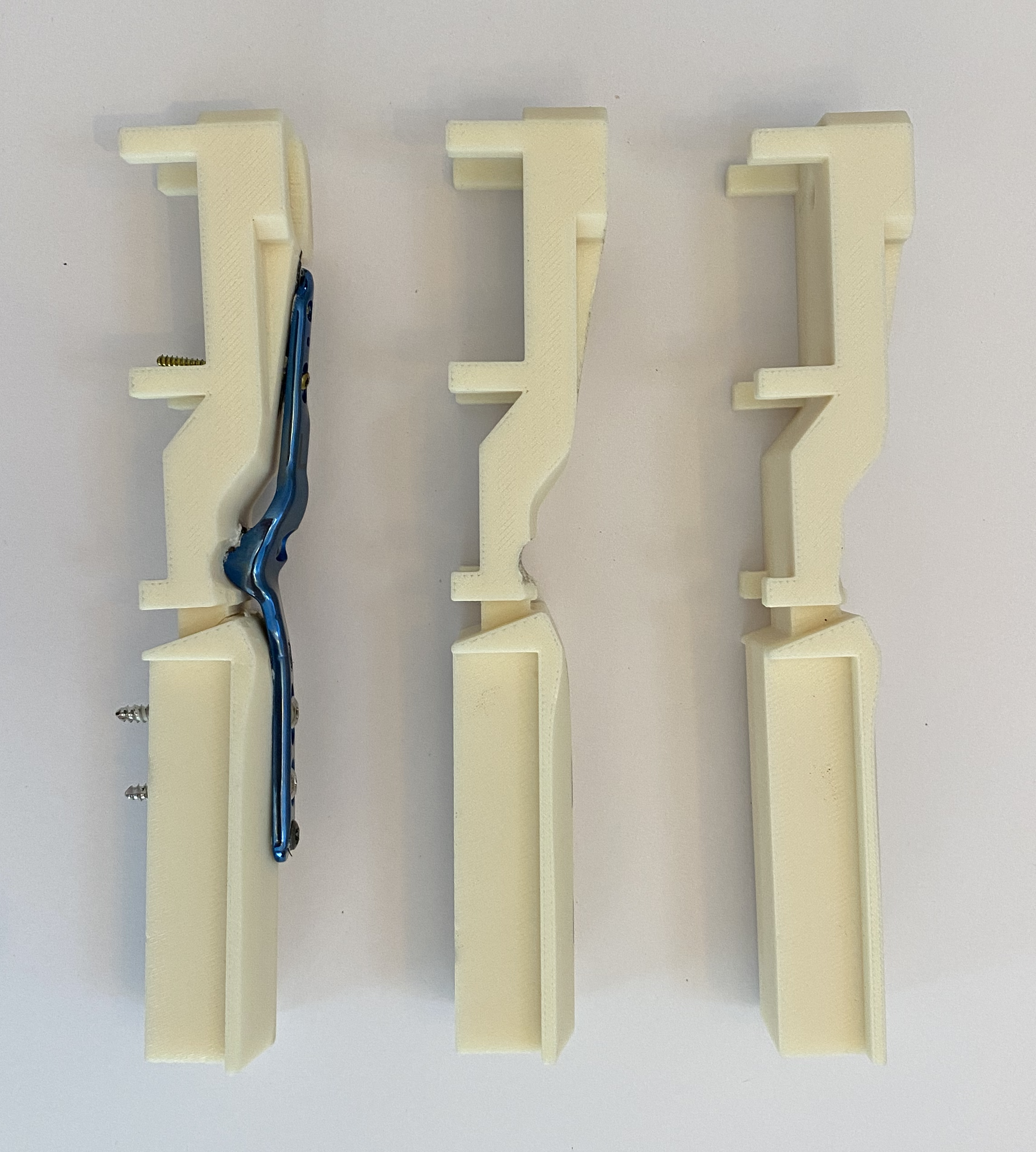


Lateral view of wrist plate model

Left: Acumed plate applied to prepared model (with CMCJ arthrodesis, shown after testing)

Middle: Model showing areas shaded in grey lead pencil that are filed away to allow the plate to sit flush on the model.

Right: Wrist model without modifications for the Acumed plate
