# Supplementary material for: Biomechanical Performance of Total Wrist Arthrodesis Plates With and Without Arthrodesis of the Carpometacarpal Joint
Source: Hand (N Y). 2023 Oct 7;20(2):230–6. doi: 10.1177/15589447231198263 (PMC11833838; doi:10.1177/15589447231198263)
Supplement: sj-docx-5-han-10.1177_15589447231198263 – Supplemental material for Biomechanical Performance of Total Wrist Arthrodesis Plates With and Without Arthrodesis of the Carpometacarpal Joint [file sj-docx-5-han-10.1177_15589447231198263.docx]

**Supplementary Material**

Supplementary Material 1: Table showing plate type with screw configuration from most distal (1) to most proximal. Wrist models with CMCJ arthrodesis and mobile CMCJ had the same screw configuration.

Supplementary Material 2: Wrist model with arthrodesis of the CMCJ (.stl files may be opened with Preview).

Supplementary Material 3: Wrist model without arthrodesis of the CMCJ (.stl files may be opened with Preview).

Supplementary Material 4: showing modification to the 3D printed wrist model to improve the fit of the Acumed plate. The Acumed plate is designed to fit to the dorsum and radial boarder of the second metacarpal. It also has an increased contour at the radial carpal joint level.

Supplementary Material 5: Video showing testing of the Synthes stainless steel (SS) plate mounted to a wrist model with arthrodesis of the CMCJ.
